# Neural Correlates of Single- and Dual-Task Walking in the Real World

**DOI:** 10.3389/fnhum.2017.00460

**Published:** 2017-09-14

**Authors:** Sara Pizzamiglio, Usman Naeem, Hassan Abdalla, Duncan L. Turner

**Affiliations:** ^1^Neuroplasticity and Neurorehabilitation Doctoral Training Programme, Neurorehabilitation Unit, School of Health, Sport and Biosscience, University of East London London, United Kingdom; ^2^School of Architecture, Computing and Engineering, University of East London London, United Kingdom; ^3^UCLPartners Centre for Neurorehabilitation, University College London London, United Kingdom

**Keywords:** mobile brain-body imaging, EEG, multitasking, neuroimaging, urban environment, gait monitoring

## Abstract

Recent developments in mobile brain-body imaging (MoBI) technologies have enabled studies of human locomotion where subjects are able to move freely in more ecologically valid scenarios. In this study, MoBI was employed to describe the behavioral and neurophysiological aspects of three different commonly occurring walking conditions in healthy adults. The experimental conditions were self-paced walking, walking while conversing with a friend and lastly walking while texting with a smartphone. We hypothesized that gait performance would decrease with increased cognitive demands and that condition-specific neural activation would involve condition-specific brain areas. Gait kinematics and high density electroencephalography (EEG) were recorded whilst walking around a university campus. Conditions with dual tasks were accompanied by decreased gait performance. Walking while conversing was associated with an increase of theta (θ) and beta (β) neural power in electrodes located over left-frontal and right parietal regions, whereas walking while texting was associated with a decrease of β neural power in a cluster of electrodes over the frontal-premotor and sensorimotor cortices when compared to walking whilst conversing. In conclusion, the behavioral “signatures” of common real-life activities performed outside the laboratory environment were accompanied by differing frequency-specific neural “biomarkers”. The current findings encourage the study of the neural biomarkers of disrupted gait control in neurologically impaired patients.

## Introduction

Bipedal walking in humans has been studied extensively from a biomechanical perspective across the healthy lifespan as well as in neurologically impaired individuals (Beyaert et al., 2015; Del Din et al., 2016). Walking is one of the most common human activities and for a significant proportion of time further tasks such as navigating, conversing or listening to music are often undertaken. However, even healthy subjects demonstrate decreased gait performance when walking and engaging in a challenging secondary task such as messaging over a smartphone (Schabrun et al., 2014; Agostini et al., 2015). For neurological impaired patients, such as hemiplegic stroke survivors or Parkinson’s disease patients (PD), walking itself can be challenging or even impossible without external support (Menz et al., 2003b; Latt et al., 2009; Iosa et al., 2014; Maidan et al., 2016). If engaged in a secondary task, even the well-recovering neurological patient demonstrates greater attention and effort accompanied with gait speed reduction and a tendency for impaired locomotion (Maidan et al., 2017). A better understanding of the neural biomarkers of gait performance in different dual tasks could shed light on the potential risks of such behaviors in healthy and neurological populations and point towards better targeted treatment approaches for improved mobility in real-world scenarios.

With progress in mobile technologies, the neural correlates of gait have been investigated recently with high-density electroencephalography (EEG; Gwin and Ferris, 2012; Wagner et al., 2012, 2014, 2016; Seeber et al., 2014; Winslow et al., 2016). Young and older adults exploit different neural and gait-postural strategies when performing a secondary task while walking, with older adults less flexible in allocating neural resources and changing neural processing strategies (De Sanctis et al., 2014; Malcolm et al., 2015). Other studies, using functional near-infrared spectroscopy (fNIRS), have observed an increase in oxygenation in the frontal brain areas during walking while performing a secondary task (Holtzer et al., 2011, 2015; Al-Yahya et al., 2016; Hernandez et al., 2016; Lin and Lin, 2016; Maidan et al., 2016, 2017). Whilst these technological developments have been used in laboratory settings, for example on treadmills or short walkways, the challenge of being able to record natural human behavior in the real-world remains (Griffiths et al., 2016; Ladouce et al., 2017).

In this study, we employed mobile brain (EEG) and body imaging (MoBI) to investigate the neural correlates of natural single- and dual-task walking in an open-space, outside the laboratory environment. In order to mimic daily-life experiences, secondary tasks consisted in either having a conversation with the experimenter or replying to an email read from a smartphone. We hypothesized first that gait performance would decrease with the engagement of a secondary task as previously described (Menz et al., 2003a; Schabrun et al., 2014; Francis et al., 2015). Second, we hypothesized that the neural correlates of single-task walking would replicate previous findings obtained within the laboratory environment (Gwin et al., 2011; Wagner et al., 2012; Seeber et al., 2014). Prediction of how the brain operates to a secondary task while walking in the real-world is challenging (Wahn and König, 2017). However, we hypothesized that real-world dual-task conditions would engage brain areas related to higher executive functions and planning (e.g., pre-frontal cortex, PFC; Ford et al., 2002; Giraud et al., 2007) and areas involved in complex sensorimotor integration and spatial navigation (e.g., sensorimotor cortex, SMC; posterior parietal cortex, PPC; Buneo and Andersen, 2006; Engel and Fries, 2010; Sipp et al., 2013; Beurskens et al., 2016; Bradford et al., 2016; Wagner et al., 2016). The results suggested that there were condition-specific patterns of neural activation that differed with the nature of the secondary task and that there were frequency-specific neural biomarkers for different real-world ambulatory scenarios.

## Materials and Methods

### Ethical Approval

Eighteen right-handed healthy young adults (mean age ± standard deviation, SD; 25 ± 3; 7 males/11 females) with no previous history of neurological, musculoskeletal or gait disorders, agreed to participate in this study by giving written informed consent. The study was approved by the University of East London Ethics Committee (UREC_1415_29) and all experiments were conducted in accordance with the Declaration of Helsinki. Data of three subjects were discarded because of problems during data acquisition (2 males/1 female) and one subject (female) was ultimately excluded from group-level analysis due to a very high level of gait-related noise within the neurophysiological data (Oliveira et al., 2017), leaving a total of 14 subjects (mean age; 26 ± 3; 5 males/9 females).

### Experimental Setup

**Figure 1:**
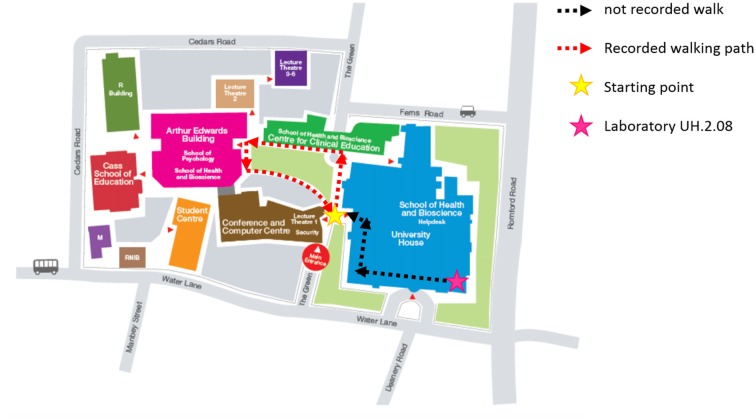
UEL Stratford Campus map and subjects walking path. Subjects were first prepared in the laboratory (pink star) and then accompanied outside along the black-dashed path. They were then given specific instructions on the path to follow during the experiment (red-dashed path), starting and finishing always in the same position (yellow start).

**Figure 2:**
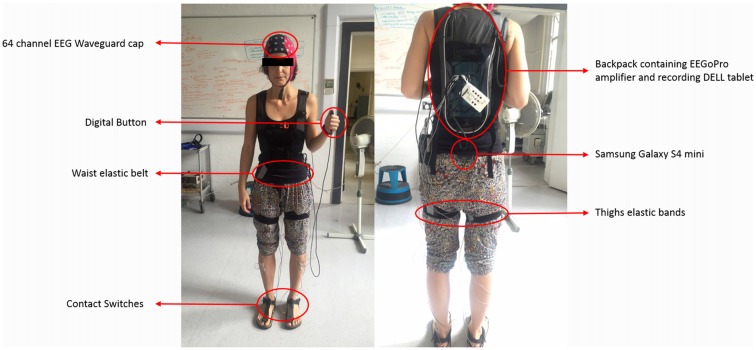
Mobile Setup for real-world experiments. During walking experiments, subjects carried all the setup on themselves. Brain activity was recorded by a 64-channel electroencephalography (EEG) Waveguard cap connected to the EEGoPro amplifier which was placed into a backpack together with a DELL tablet on which the recording software ran. Contact switches were placed underneath the subject’s heels and connected to a digital input of the MWX8 DataLog analog-to-digital converter. The converter was fixed at the subject’s hips level by an elastic belt. Elastic bands placed around the subject’s thighs made sure cables remained fixed and did not disturb the gait performance. A digital button was also connected to the converter through a secondary digital input and eventually pressed by the subject at specific time points. The Samsung Galaxy S4 mini was firmly placed at the subject’s lower back through the elastic belt. The author SP gave informed consent for the publication of this image.

Subjects were first prepared in a laboratory room (Figure 1). Once ready, they started the experiment by performing 3 min of resting standing still (i.e., Baseline) with their eyes open looking at a standard spot on a blank wall. Subjects were then guided through the building outside to the garden (Figure 1). During this period, no signals were recorded and subjects were instructed to get familiar with the setup and communicate to the experimenter if anything was not properly set. Once outside, subjects were given all the details related to the experiment, specifically: (1) the predefined walking path was shown to them (Figure 1); (2) they were instructed to walk at their preferred natural speed, as it has been shown that this optimizes gait behavior (Sekine et al., 2013); and (3) they were asked to minimize any extreme movements that could have affected the recordings. Experiments consisted of three conditions during which subjects walked along the predefined path without engaging in any secondary task (single-task walking, ST), conversing with the experimenter (dual-task1 walking, DT1) or texting with their smartphone (dual-task2 walking, DT2). The dual-task conditions were randomized across subjects in order to avoid bias in gait behavior and recordings. The dual-task conditions were designed to represent real-life situations and to standardize them, conversations during DT1 were based on a set of standard questions, whereas in DT2 subjects read and replied to a standard email. In each condition, subjects walked the predefined path twice covering a total distance of 200 m. Resting periods were given to subjects between conditions to avoid fatigue and to remind them of the instructions for the next condition. Experiments were carried out only during dry days free from strong winds and/or rain.

### Recording Techniques

The implemented setup presented in Figure 2 is fully mobile and allows the recording of physiological and behavioral data during walking (or any other mobile situation). Brain activity (EEG; μV) was recorded via a high-density 64 channel Waveguard cap (ANT Neuro, Enschede, Netherlands), with impedances kept below 5 kΩ for the whole duration of the experiment. EEG activities were continuously recorded by an EEGoPro amplifier (ANT Neuro, Enschede, Netherlands) at a sampling frequency of 1 kHz. During the recording, EEG data were referenced to the FCz channel. Data were recorded and saved by the EEGoPro software installed on a PC-tablet, connected via USB to the amplifier and carried by the subject within the backpack, together with the amplifier. A Samsung Galaxy S4 mini smartphone was fixed at the subject’s lower back with an elastic belt and data from its internal accelerometers and gyroscope were recorded through the AndroSensor app^1^ at a frequency of 200 Hz, saved as .csv files at the end of each condition and ultimately downloaded for offline analyses. The lower back position is currently the most preferred and reliable location to observe changes in gait patterns across different conditions and populations (Iosa et al., 2014). Two digital force sensing resistor sensors (FSRs) were employed as contact switches and fixed underneath the subject’s heels to detect times of heel strikes. Data were recorded at 1 kHz by a 14 bit analog-to-digital converter (DataLog MWX8, Biometrics Ltd, Newport, UK) fixed at the subject’s hip by the elastic belt. A digital button (1-to-0 active edge) was also connected to the converter and pressed by the subject for *circa* 5 s at the beginning and at the end of each condition to define time points of start and finish. Elastic bands were also placed around the subject’s thighs to fix the cables and prevent the subject from falling/stepping on them. To synchronize data from the digital sensors representing important time points (i.e., start, heel strikes, end) with physiological variables, a common train of 12 consecutive TTL pulse was simultaneously sent to both the DataLog MWX8 converter and the EEGoPro amplifier at the beginning and at the end of the experiment, in order to be able to detect and correct for drifts in the time over the recordings. The matching of start and end points of each single pulse between the recordings were checked offline and eventually used as milestones for realigning the signals’ time axes. Moreover, a video of the subject walking during each condition was recorded to monitor subject behavior and keep track of any important events (e.g., external disturbance, big movements, etc.).

### Data Analysis

Offline data analyses were run in MatLab 2015b (The MathWorks, Inc., Natick, NA, USA). First of all, the time of the first TTL pulse was detected in both the digital and the physiological recordings in order to synchronize the data. Second, time points of each button press were identified to divide the continuous recordings into conditions (ST, DT1 and DT2). Last, time points of each heel strike were extracted and related events were created in the physiological data file. From these latencies, measures of gait performance were also evaluated for later use.

#### Gait Measures

Linear acceleration data recorded in each condition with the smartphone were separately uploaded into the free software iGAIT (MatLab interface) for the analysis of gait performance (Yang et al., 2012). Several spatio-temporal as well as frequency features of gait were extracted, for example mean step length (m), step cadence (step/min), gait velocity (m/s), stride regularity (a.u.) and gait acceleration as root mean square (RMS) in each movement direction (i.e., Vertical (ver-), Medio-Lateral (ml-), Antero-Posterior (ap-)). Acceleration RMS is a measure of the magnitude of the acceleration (i.e., trunk movements) in each movement direction and has been extensively applied in the evaluation of gait abnormalities in healthy subjects as well as neurological patients (Latt et al., 2009; Iosa et al., 2014; Van Criekinge et al., 2017).

#### EEG Pre-Processing

Offline pre-processing of EEG data was carried out using EEGLab toolbox for MatLab (Delorme and Makeig, 2004). Data were first band-pass filtered between 0.5 Hz and 100 Hz to minimize slow drifts and remove high-frequency components and notch filtered at 50 Hz to remove the power line noise. Visual inspection was performed on continuous data, where EEG channels affected by major noise sources throughout the whole experiment were identified and temporarily removed from the analyses. Prominent artifacts affecting all the recording channels were also removed from the data. Data were then re-referenced to the common average reference and decomposed using Independent Component Analysis (ICA) with the extended Infomax algorithm as implemented in EEGLab (Cardoso, 1997; Delorme et al., 2007). Power spectral, spatial and temporal features of each independent component (IC) were carefully inspected and those representing typical artifacts (e.g., eye blinks, saccades, neck muscles activity) were removed from the data. Remaining components were projected back to the scalp channels, previously removed bad channels were interpolated and all data then re-referenced again to the common average reference. Continuous data were then segmented into epochs of 1.8 s duration from −200 ms to 1600 ms around each right heel strike in order to capture a complete stride (composed by, in order: right, left, right heel strikes) even at the slowest speed. A final visual inspection was performed to check the quality of the cleaned data and eventually remove still noisy epochs.

#### Time-Frequency Analyses

##### Time-frequency analysis with Morlet wavelet decomposition

Time-frequency analysis was performed with functions provided by the EEGLab toolbox (Makeig, 1993; Delorme and Makeig, 2004). The spectral power changes with respect to the log-spectrum of a baseline period were evaluated in each epoch using Morlet wavelet decomposition (high frequency: 50 Hz; wavelet width at lowest frequency: 3 oscillation cycles; wavelet width at highest frequency: 14.35; Hanning window size: 350 ms; Time steps: 10 ms). Additional information on the choice of these parameters are reported in Supplementary Material (see Section S1, Supplementary Figures S1–S3). Two different baseline approaches were used following previous work (Wagner et al., 2012; Seeber et al., 2014), first, the log spectrum of the 3 min period of resting state standing still with eyes-open was used; then second, the mean gait cycle log spectrum was employed. Single-epoch spectrograms were first computed and time warped to the median step latency (across subjects) using linear interpolation (Gwin et al., 2011; Wagner et al., 2012). With this method, time points of heel strikes in each epoch were aligned across epochs. Spectral power changes with respect to the baselines were evaluated through the mean difference between each single-epoch log-spectrum and the mean baseline log-spectrum. This methodology was employed in order to obtain an informative visualization of each subject power spectrum during one full stride in each condition and check the quality of the pre-processed data. Group-level significant changes from the mean baseline (i.e., either resting-state either mean gait cycle) log-spectrum were computed through the bootstrapping method with FDR correction for multiple comparisons (*p* < 0.05) according to previous work (Wagner et al., 2012, 2016).

##### Power spectral density (PSD)

Each condition was considered as *continuous* as no external triggers were employed and subjects performed the same task for the whole condition duration. To assess spectral information regardless of the time domain, for each subject, separately for each condition (also for the resting-state) and for each electrode, the Power Spectral Density (PSD) was measured in each epoch through the Welch’s overlapped segment averaging estimator. A default Hamming window of 400 ms with a 50% overlap (i.e., 200 ms) was adopted and PSD for frequencies from 2 Hz to 50 Hz was calculated.

### Statistics

Statistical analyses were run in SPSS 23 software (IBM). EEG specific statistical analyses were run in MatLab 2015b using methods implemented in FieldTrip (Maris and Oostenveld, 2007).

#### Statistical Analyses of Gait Measures

Gait measures were first assessed separately for each condition in each subject and ultimately group-level differences between conditions were assessed. Kolmogorov-Smirnoff test for normality was first used to test the distribution of the data. Data were all normally distributed, thus parametric statistical tests where employed. One way repeated measures analysis of variance (ANOVA) with factor “Condition” (three conditions) was applied to each gait measure of interest to identify significant variance across conditions. Greenhouse-Geisser adjustments were employed when appropriate. Subsequently, paired samples *t*-tests with Bonferroni correction for multiple comparisons were run to specifically define differences between conditions. Significance level was set at *α* = 0.05, with number of repeated measures = 3 (ST vs. DT_i_ with *i* = 1, 2 and DT1 vs. DT2), which meant an adjusted *α* = 0.05/3 = 0.0167 for multiple comparisons.

#### Non-Parametric Cluster-Based Permutation Test on PSD

Differences of sensor-level PSD across conditions were assessed through non-parametric cluster-based permutation tests as provided in FieldTrip. This analysis has been extensively used in EEG studies as it successfully tackles the multiple comparisons problem (MCP; Maris and Oostenveld, 2007; Negrini et al., 2017). Specifically, a paired sample *t*-Test was conducted for each electrode and *t*-values exceeding an “*a priori*” threshold were clustered based on adjacent neighboring electrodes. Cluster-level statistics were computed by taking the sum of the *t*-values within every cluster. The statistical comparisons were performed with respect to the maximum values of summed *t*-values. By means of a permutation test (i.e., randomizing data across conditions and rerunning the statistical test *N* times) the distribution of the maximum of summed cluster *t*-values was obtained and further employed to evaluate the statistics of the actual data. Clusters in the original dataset were considered to be significant at an alpha level (α_cluster_) of 1% if less than the 5% of the permutations (α_cluster_ = 0.01, *α* = 0.025 for two-tailed tests, *N* = 1500) used to construct the reference distribution yielded a maximum cluster-level statistics larger than the cluster-level value observed in the original data. A positive cluster represents a statistically significant increase of activity in the first term of one comparison with respect to the second term. A negative cluster represents a statistically significant decrease of activity in the first term of one comparison with respect to the second term. Cluster-based permutation tests were run on PSD data for each frequency of interest (FOI) separately, specifically θ (4–7 Hz), α (8–12 Hz) and β (15–30 Hz) for three different tests (ST vs. DT_i_ with *i* = 1, 2 and DT1 vs. DT2). Further correction for multiple comparisons was run with the Bonferroni method (*p* = 0.025/*9* = 0.0028 for two-tailed test). All the channels were simultaneously entered in the analysis.

## Results

### Gait Measures

**Figure 3:**
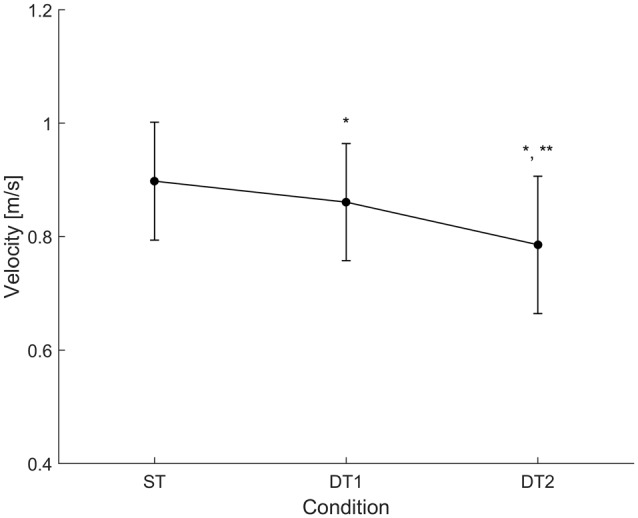
Condition-by-condition gait velocity. A condition-by-condition population average (*N* = 14) profile with standard deviation error bars. Average gait velocity decreases in the two dual-task conditions with respect to the single-task condition. Statistically significant paired-samples *t*-test corrected for multiple comparisons (Bonferroni, ×3) are highlighted with *(ST vs. DT*_i_* with *i* = 1, 2) and/or **(DT1 vs. DT2). Detailed results are reported in Table 1.

Subjects (*N* = 14) walked along the predefined path of 200 m in an average time of 225 ± 25 s during single-task (ST) walking, and significantly more slowly when walking while conversing (DT1; 235 ± 28 s) and when walking while texting (DT2; 260 ± 41 s); RMANOVA *F* = 21.660, *p* < 0.001). Similar changes in gait velocity were observed (Figure 3). Descriptive statistics for all gait measures across the three conditions are reported in Table 1. Velocity as the only measure significantly different (ANOVA df = 2, *F* = 34.215, *p* = 0.001) in both DT1 (df = 13, *t* = 4.199, *p* = 0.001) and DT2 (df = 13, *t* = 6.847, *p* = 0.001) with respect to the ST condition, and between DT1 vs. DT2 (*t* = 4.991, *p* = 0.001). Gait measures of ver-RMS and ap-RMS, whose ANOVA was significant (df = 2, *F* > 16.946, *p* < 0.001), only showed statistical differences between DT2 vs. ST (df = 13, *t* < −3.503, *p* < 0.004) and DT2 vs. DT1 (df = 13, *t* < −3.793, *p* < 0.002). Cadence, ml-RMS and ap-stride regularity, whose ANOVA was significant (1.205 < df < 1.426, *F* > 4.559, *p* < 0.043), only showed statistical differences between DT2 vs. ST (df = 13, *t* > 2.772, *p* < 0.016). The gait measure of ml-stride regularity, whose ANOVA was significant (df = 2, *F* = 3.990, *p* = 0.031), only showed statistical differences between DT2 vs. DT1 (df = 13, *t* = 3.618, *p* = 0.003). No significant changes in mean step length and ver-stride regularity were detected across conditions.

**Table 1:** Single- and Dual-task conditions gait measures.

	Single Task	Dual Task 1	Dual Task 2	Anova df	Anova *F*	Anova *p*
Cadence (step/min)	107 (± 10)	104 (± 12)	99 (± 16)*	1.205	4.559	0.043
Velocity (m/s)	0.90 (± 0.10)	0.86 (± 0.10)*	0.78 (± 0.12)*,**	2	34.215	0.001
Mean step length (m)	0.53 (± 0.06)	0.52 (± 0.08)	0.51 (± 0.07)	2	0.0769	N.S.
ver-RMS	2.65 (± 0.56)	2.59 (± 0.55)	2.26 (± 0.63)*,**	2	17.554	0.001
ml-RMS	1.48 (± 0.32)	1.47 (± 0.31)	1.37 (± 0.40)*	1.339	7.769	0.008
ap-RMS	2.19 (± 0.28)	2.09 (± 0.25)	0.96 (± 0.33)*,**	2	16.946	0.001
ver-stride regularity	0.75 (± 0.07)	0.68 (± 0.19)	0.65 (± 0.16)	2	2.537	N.S.
ap-stride regularity	0.72 (± 0.08)	0.69 (± 0.10)	0.65 (± 0.12)*	2	7.158	0.009
ml-stride regularity	0.46 (± 0.20)	0.46 (± 0.13)	0.35 (± 0.16)**	1.426	3.990	0.031

Condition-by-condition (n = 3) mean (± SD) of the measures of gait of interest for the sample population (N = 14). Repeated measures ANOVA p-values are reported in the sixth column on the right. Statistically significant paired-samples *t-test* corrected for multiple comparisons (Bonferroni, ×3) are highlighted with * (ST vs. DT_i_ with i = 1, 2) and/or ** (DT1 vs. DT2).

### Neurophysiological Measures

**Figure 4:**
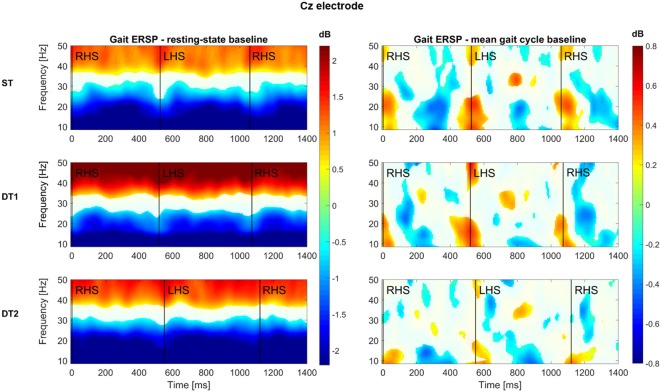
Group-level time-frequency analyses across conditions for Cz electrode. All subjects (*N* = 14) average time (x axis)—frequency (y axis) representations of the spectral power of the Cz electrode are here reported for each condition (first row: single-task walking; second row: dual-task1 walking; third row: dual-task2 walking). Two baseline approaches have been used: on the left hand side, the log spectrum of a 3 min period of resting state standing still with eyes-open was used; on the right hand side, the mean gait cycle log spectrum was employed. Color-bars (dB) are constant across conditions within each baseline approach and report increase (values >0, warm-color coded) and decrease (values <0, cold-color coded) of power spectrum with respect to each specific baseline. Group-level significance was calculated via bootstrapping method and FDR correction for multiple comparisons (*p* < 0.05) according to Wagner et al. (2012). A white mask was applied on those time-frequency bins (i.e., pixels) that did not pass the statistical test.

Figure 4 shows the results of the group-level time-frequency analysis with Morlet wavelet spectral decomposition across the three conditions (epochs averaged: ST = 179 ± 28, DT1 = 148 ± 20, DT2 = 188 ± 30) for the electrode Cz (i.e., located above the motor cortex area of legs/feet; Wagner et al., 2012) with values expressed in dB. When using the resting-state as the baseline, a sustained α (8.4–12 Hz) and β (15–30 Hz) desynchronization (color coded in blue) is clear for the whole stride duration and consistent across conditions. At the same time, a synchronization (color coded red) is visible at higher frequencies, from 30 Hz to 50 Hz. When using the mean gait cycle as the baseline, amplitude modulations occurred related to the mean gait cycle and time locked to the gait cycle dynamics. Specifically, augmented α and β power is visible at the end of stance phase (i.e., when the leading foot is in contact with the ground), whereas a decrease of power in these two frequency bands happens in between stance phases. Similar patterns are illustrated across conditions, although the DT2 condition shows reduced significant modulations.

**Figure 5:**
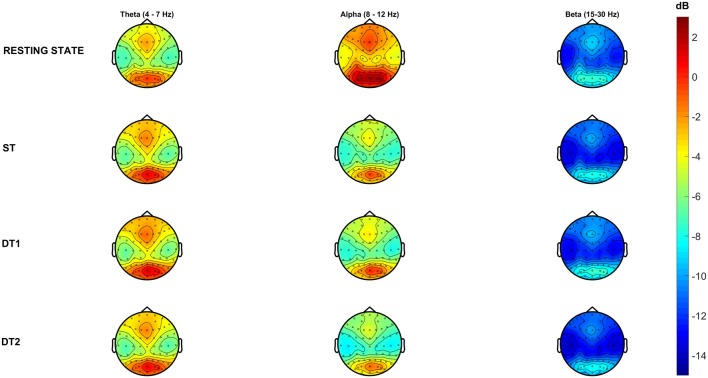
Grand-average Power Spectral Density (PSD) across conditions in each frequency of interest (FOI). Topographical representations of all subjects (*N* = 14) average PSD across conditions describes high (warm-color coded) and low (cold-color coded) intensities of PSD (color-bar (dB) is constant across conditions and frequency bands).

As talking implies the activation of facial muscles and mouth movements to create speech, substantial artifactual muscular activations might interfere with brain related signal sources, despite the careful pre-processing pipeline. Therefore, high frequency brain oscillations (>30 Hz) will not be considered in subsequent analyses. Figure 5 shows topological representations (i.e., topoplots) of the PSD grand-average (number of epochs averaged: RS = 96 ± 7, ST = 179 ± 28, DT1 = 148 ± 20, DT2 = 188 ± 30) across all subjects for the three main FOIs: θ (4–7 Hz), α (8–12 Hz) and β (15–30 Hz).

### Single-Task Walking vs. Resting State EEG—Cluster-Based Permutation Tests on PSD

**Figure 6:**
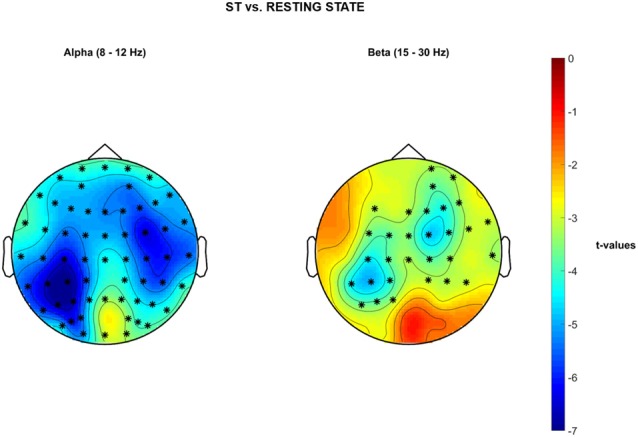
Non-parametric cluster-based permutation test comparing PSD in ST vs. Baseline resting state standing still with eyes open. Topographical maps are color-coded according to the permutation tests *t*-values resulted from the comparison of PSD between single-task walking (ST) and Resting-State (i.e., Baseline). Clusters of electrodes whose PSD is significantly different between the two conditions are highlighted in *(*p* < 0.002 after Bonferroni correction). In the α frequency band, a general decrease of PSD activity is reported over the whole brain during single-task walking with respect to baseline. In the β frequency band, a decreased PSD activity occurs in a wide cluster including right-frontal-, bilateral-central- and bilateral parietal areas during single-task walking in comparison to baseline.

Significant cluster-based permutation tests *t*-values topological maps are reported in Figure 6 for the comparison ST vs. resting EEG. A significant decrease of PSD in the α band over the whole brain was reported during ST1 in comparison to resting-state standing still, with stronger differences (i.e., deeper blue color = lower *t*-values) bilaterally over the sensorimotor cortices (negative cluster = all electrodes, *p* = 0.001). Moreover, a significant decrease of PSD in the β band was observed in a wide cluster including electrodes located over frontal-, central- and parietal- bilateral areas during ST1 with respect to resting-state standing still (negative cluster = {FP2, F3, FZ, F4, FC1, FC2, FC6, C3, CZ, C4, T8, CP5, CP1, CP2, CP6, P3, AF4, AF8, F2, F6, FC3, FCZ, FC4, C1, C2, CP3, CP4, P5, P1, FT8}, *p* = 0.002). No significant differences were detected for θ PSD.

### Dual-task vs. Single Task Walking EEG—Cluster-Based Permutation Tests on PSD

**Figure 7:**
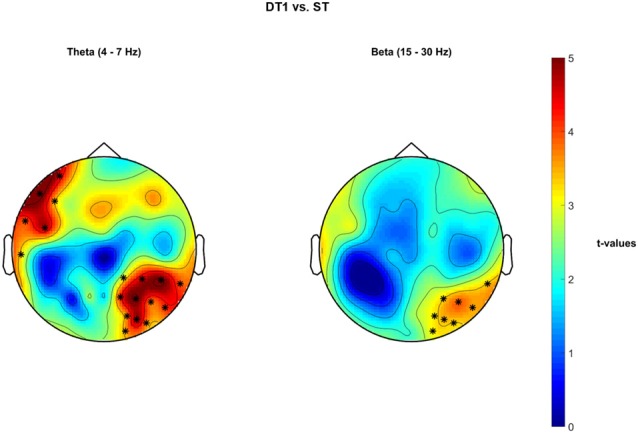
Non-parametric cluster-based permutation test comparing PSD in DT1 vs. ST. Topographical maps are color-coded according to the permutation tests *t*-values resulted from the comparison of PSD between dual-task1 walking (DT1) and single-task walking (ST). Clusters of electrodes whose PSD is significantly different between the two conditions are highlighted in *(*p* < 0.002 after Bonferroni correction). In the θ frequency band, an increased PSD activity occurs in a left frontal and in a right occipital-parietal cluster of electrodes during DT1 with respect to ST. In the β frequency band, an increased PSD activity occurs in a right occipital-parietal cluster of electrodes during DT1 in comparison to ST.

Significant cluster-based permutation tests *t*-values topological maps are reported in Figure 7 for the comparison DT1 vs. ST. Specifically, a significant increased PSD in the θ band occurred in a right-parietal-temporal cluster of electrodes and in a left-frontal-temporal sensor cluster (positive cluster 1 = {CP2, CP6, P4, P8, O2, CP4, P2, P6, PO4, PO6, TP8, PO8}, *p* = 0.002; positive cluster 2 = {T7, F7, FC5, AF7, F5, FT7}, *p* = 0.002). Moreover, a tendency towards a significant increase of PSD was observed in the β band in a right-parietal cluster of electrodes (positive Cluster = {P4, P8, O2, P6, PO4, PO6, TP8, PO8}, *p* = 0.005). No statistically significant differences were obtained for the comparison DT2 vs. ST.

**Figure 8:**
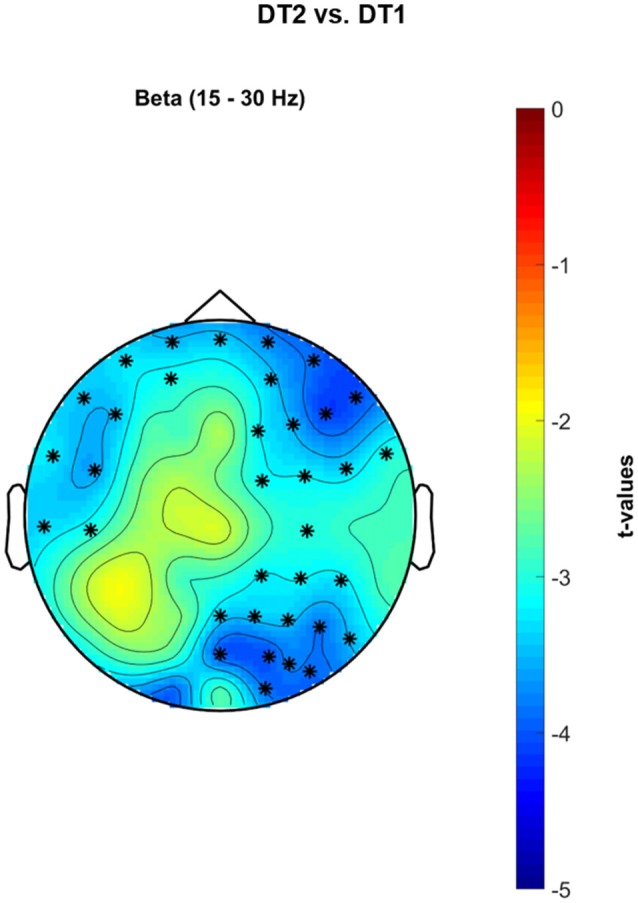
Non-parametric cluster-based permutation test comparing PSD in DT2 vs. DT1. Topographical maps are color-coded according to the permutation tests *t*-values resulted from the comparison of PSD between dualtask2 walking (DT2) and single-task walking (ST). Clusters of electrodes whose PSD is significantly different between the two conditions are highlighted in *(*p* < 0.002 after Bonferroni correction). In the β frequency band, a decreased PSD activity occurs in a wide cluster of electrodes extending from left-central frontal-temporal regions to right occipital-parietal areas during DT2 with respect to DT1.

Figure 8 shows significant cluster-based permutation tests *t*-values topological maps for the comparison DT2 vs. DT1. A significant decrease of PSD occurred in the β band in a wide frontal-premotor, and right-sensorimotor cluster of electrodes (negative cluster = {FP1, FPZ, FP2, F7, F4, F8, FC5, FC2, FC6, T7, C4, CP2, CP6, PZ, P4, P8, POZ, O2, AF7, AF3, AF4, AF8, F5, F2, F6, FC4, C5, CP4, P2, P6, PO4, PO6, FT7, FT8, PO8}, *p* = 0.002).

## Discussion

### Novel Findings

The current study suggests that gait performance (as described with measures of velocity and intensity of trunk movements) decreased with increasing task demands and challenges in line with previous literature (Menz et al., 2003a,b). In parallel, brain activations over premotor, motor and parietal regions demonstrated activation patterns specific to walking (see Figures 4, 6) in agreement with the current literature (Gwin et al., 2011; Wagner et al., 2012, 2014, 2016; Seeber et al., 2014; Bradford et al., 2016). Previous studies on the neural correlates of multitasking during walking have focused mostly on the PFC only because of technical limitations of fNIRS (Al-Yahya et al., 2016; Maidan et al., 2017). However, we demonstrated that other brain areas such as the posterior parietal brain regions are involved during different dual-task conditions and specifically recruited by the brain according to possible attentional and energy-optimization strategies (see Wahn and König, 2017). Additional analyses demonstrated that residual artifacts from possible facial and neck muscle activity did not affect the results (see Section S2, Supplementary Figures S4–S7).

### Gait Measures

A decrease in gait velocity and regularity (i.e., stride regularity) was related to increased task-related motor and cognitive demand. Previous studies reported changes in gait pattern when simultaneously talking (Holtzer et al., 2011) or texting (Schabrun et al., 2014; Agostini et al., 2015). Both secondary tasks induced a decrease of gait speed, with the latter also impairing gait stability. This may be attributed to the fact that people are more used to walking while talking than they are to walking while texting. Previous studies have investigated the relationship between dual-task demand and gait behavior with the aim of identifying which elements mostly undermine performance. Cognitive interferences such as mental arithmetic (Springer et al., 2006; Francis et al., 2015) and sensorimotor tasks (Beurskens and Bock, 2013) seem not to affect gait performance as much as motor interferences such as road complexity (Menz et al., 2003a,b; Lin and Lin, 2016; Maidan et al., 2016) and hand engagement (Beurskens et al., 2016). Secondary tasks that require a higher continuous visual processing have been classified as more likely to impair gait performance (Beurskens and Bock, 2013; Francis et al., 2015). Indeed, a continuous scan of the surrounding environment is crucial when walking on difficult paths (Matthis et al., 2017). Walking and texting prevent subjects from monitoring the surroundings as their eyes are mostly focused on the phone, thus decreasing the visual scan of the environment and altering the gait performance. This does not hold for walking and talking for example, during which subjects can continuously (or periodically, if sometimes looking at the speaker) scan the surrounding environment thus maintaining gait stability and performance. Gait deficits related to lack of constant visual processing are indeed exacerbated in people with reduced executive functions capabilities such as the elderly with a history of falls (Springer et al., 2006). If lack of visual processing skills is the major contributor to gait instability during dual-task walking in health, then other neurological impairments could undermine gait performance *per se* as a consequence of the neural injuries. Elderly (Holtzer et al., 2011; Iosa et al., 2014), PD patients (Latt et al., 2009; Maidan et al., 2016) and stroke survivors (Al-Yahya et al., 2016) always demonstrate reduced performance when task difficulty increases. Conversely, healthy young adults usually show little to moderate changes in gait behavior when simultaneously performing secondary tasks and this could be related to their more effective adaptive strategies and mechanisms.

### Neurophysiological Measures

Previous investigations have questioned whether dual-tasking significantly alters brain activity, which elements influence these changes the most and which populations are most affected by reduced multi-tasking abilities. fNIRS over the pre-frontal cortex has been widely used for testing these hypotheses. Different groups have found consistent increases in oxygen levels in the pre-frontal cortex of healthy young adults when performing any type of dual-task condition (Holtzer et al., 2015; Al-Yahya et al., 2016; Lin and Lin, 2016) and the increases are greatest in those populations who are more cognitively impaired such as the elderly, stroke patients and PD patients (Holtzer et al., 2011; Al-Yahya et al., 2016; Maidan et al., 2017). These studies confirmed the active recruitment of the prefrontal cortex during multi-tasking, but were limited by the restricted number of channels that could be recorded (i.e., only pre-frontal cortex, no possibility for whole-brain recordings) and by the time-resolution of the activity recorded (i.e., oxygen levels changing over a time period of several seconds). Recent investigations employed high-density EEG to shed some light on the changes in more widespread neural prioritization strategies underlying cognitive performance while walking (De Sanctis et al., 2014; Malcolm et al., 2015). Healthy young adults performing a Go/NoGo inhibition task while walking showed similar cognitive performance with respect to when seated, even though their average stride duration increased and stronger and earlier frontal activations occurred. These observations suggest the engagement of more cognitive demanding processing strategies (De Sanctis et al., 2014). On the other hand, older adults who engaged in the same task maintained a stable gait behavior and neural activations with however increased dual-task cost in the cognitive domain. This may resemble a postural prioritization strategy for safe walking as well as a less flexible capability to re-allocate cortical resources (Malcolm et al., 2015). These studies support the argument that real-world investigations are needed to provide further information on the actual neural strategies underlying everyday multitasking situations.

#### Single-Task Walking in the Real-World

Single subject brain activities registered over the sensorimotor areas during single-task walking (ST) showed sustained α and β desynchronization throughout the gait cycle duration in parallel with a gait-cycle specific modulation at higher frequencies (Figure 4, left and right sides respectively; Gwin et al., 2011; Seeber et al., 2014; Wagner et al., 2014). At the group level (Figures 5, 6), α and β frequency band desynchronization is strong over the two sensorimotor areas in line with previous studies (Seeber et al., 2014; Wagner et al., 2014). Evidence of bilateral sensorimotor activations during lower limb joints movements have been previously reported in fMRI investigations with healthy adults (Kapreli et al., 2006) and stroke survivors (Enzinger et al., 2009). Moreover, recent mobile EEG studies have shown cortical activations both in medial and bilateral sensorimotor regions when walking on a treadmill (Gwin et al., 2011; Wagner et al., 2012), even at different gait speeds (Bulea et al., 2015). The bilateral activations (see 5) could be also enhanced by the active engagement of the arms swinging while walking as previously shown in literature (Miyai et al., 2001) or holding the phone and typing with the fingers. As previously suggested in the literature (Wagner et al., 2012, 2014; Ehinger et al., 2014), α and β desynchronization represent an “active state” of the brain and are likely to be involved in maintenance of the current motor status that promotes the voluntary movement of walking (Engel and Fries, 2010). α PSD is lower over the whole brain during single-task walking with respect to the resting-state recorded standing still. The peak neural activity over posterior-occipital areas (see Figure 6, yellow area) is likely to be involved in visual scanning and processing of inputs from the environment (Wagner et al., 2014).

#### Walking While Conversing

Walking while talking to a friend is one of the most common dual-task activities people perform in their daily-life. A significantly higher θ theta activity was observed in a cluster of electrodes located over the left frontal-temporal cortex, which could be associated to an increased activity of the Broca area for the creation of speech. Moreover, a second group of channels located over the right posterior parietal-occipital cortex (see Figure 7) showed significantly higher θ and β activities. Intracranial studies on monkeys performing a visual search attention task showed an active involvement of β oscillatory activity in frontal and parietal regions during top-down attention (Buschman and Miller, 2007). Neurological impairments, such as neglect, could cause a reduction of frontal-parietal network strength within the θ and β frequency bands during conscious visual tasks (Yordanova et al., 2017), validating the hypothesis of their involvement in both spatial attention and visual processing. On the other hand, increased θ activations in both prefrontal and medial-temporal lobe has been previously shown to positively correlate with successful recall of encoded words (Sederberg et al., 2003), with successful decision making regardless of spatial learning (Guitart-Masip et al., 2013), and with orchestrating item distinction, verbal working memory and long-term memory (Meyer et al., 2015). Studies on speech detection, understanding and creation demonstrated the active involvement of right temporal θ activity (Giraud et al., 2007). Frontal and temporal θ oscillatory activity was also reported in studies of schizophrenia and further linked to the attribution of inner thoughts to the external voice (Ford et al., 2002). We therefore suggest that, during the DT1 condition, stronger activations were recorded from electrodes located over the left pre-frontal and the right temporal-parietal-occipital areas as they are likely recruited as a top-down attentional mechanism to simultaneously orientate through space, listen and understand the posed questions, recollect the correct memories and eventually create speech (Simons and Spiers, 2003; Giraud et al., 2007).

#### Walking While Texting with the Smartphone

Walking while texting with a smartphone can be commonly observed now and occurs in the most complex situations such as crossing a crowded road or stepping on/off the train. When walking and simultaneously texting, the healthy young population sample recruited in our study had reduced gait speed and trunk movements (as expressed through measures of acceleration RMS). There was a stronger β desynchronization in DT2 with respect to DT1 in a broad cluster of electrodes encompassing the left motor/premotor regions, the bilateral prefrontal and frontal cortex and the right sensorimotor and parietal cortex (see Figure 8). β desynchronization represents an “active state” of the brain during which sensorimotor integration is promoted to maintain the ongoing voluntary movement (Buneo and Andersen, 2006; Engel and Fries, 2010). More challenging motor or secondary tasks have been shown to induce an even stronger β desynchronization as a basis for stronger sensorimotor integration, performance maintenance and error monitoring (Sipp et al., 2013; Bulea et al., 2015; Beurskens et al., 2016; Bradford et al., 2016; Wagner et al., 2016). Indeed, the most challenging dual-task paradigms for both healthy young adults and neurologically impaired populations are those in which visual scanning of the external environment is prevented or altered (Matthis et al., 2017). Visual scanning time was restricted during DT2 in this study (i.e., subjects were looking at the smartphone screen for a significant amount of time whilst walking). It is also likely that motor areas were activated more widely as the upper limbs, fingers and hands were engaged in typing on the phone. These elements lead to the conclusion that a stronger sensorimotor integration, as expressed in terms of stronger β desynchronization, is needed for maintaining gait stability and spatial navigation as well as performing the secondary cognitive and manual task.

#### Limitations and Future Perspective

MoBI is a novel field of research investigating human behavior in the natural environment (Makeig et al., 2009; Gramann et al., 2014, 2017; Ladouce et al., 2017). The biggest challenge is defining measurable data and comparing data to that collected in controlled laboratory-based investigations. In the current study, subjects walked naturally during the single-task condition but were free to think about anything. Moreover, in the urban environment there are many external stimuli that could have captured the subjects’ attention and impacted on their engagement in the tasks employed in the current study. Therefore, future studies should compare the level of engagement in each single task separately and in both tasks simultaneously in order to properly disentangle attention allocation and consequent impact on behavior and performance. Lastly, the analyses performed in the current investigations were restricted to the sensor-level (i.e., electrodes data), from which direct neurophysiological inferences cannot be made. Future studies should therefore aim to reliably localize (with subject-specific MRI and EEG electrodes positions) sources of neural activation and the frequency/phase coupling among them during real-world daily-life situations.

## Author Contributions

SP: study concept and design, data acquisition, data analysis and interpretation, statistical analysis, drafting/revising the manuscript for content. DLT: study concept and design, data interpretation, drafting/revising the manuscript for content. HA and UN: study concept, drafting/revising the manuscript for content. All the authors revised the final version of the manuscript.

## Conflict of Interest Statement

The authors declare that the research was conducted in the absence of any commercial or financial relationships that could be construed as a potential conflict of interest.

## Footnotes

^1^http://www.fivasim.com/androsensor.html
