# Supplementary material for: Neural Correlates of Single- and Dual-Task Walking in the Real World
Source: Front Hum Neurosci. 2017 Sep 14;11:460. doi: 10.3389/fnhum.2017.00460 (PMC5603763; doi:10.3389/fnhum.2017.00460)
Supplement: Supplementary file 1 [file Data_Sheet_1.docx]

Supplementary Material

**Neural correlates of single- and dual-task walking in the real world**

**Sara Pizzamiglio, Usman Naeem, Hassan Abdalla, Duncan L. Turner^*^**

***Correspondence:**Prof. Duncan L. Turner
[d.l.turner@uel.ac.uk](mailto:d.l.turner@uel.ac.uk)

**S1 - Time-frequency resolution trade-off**


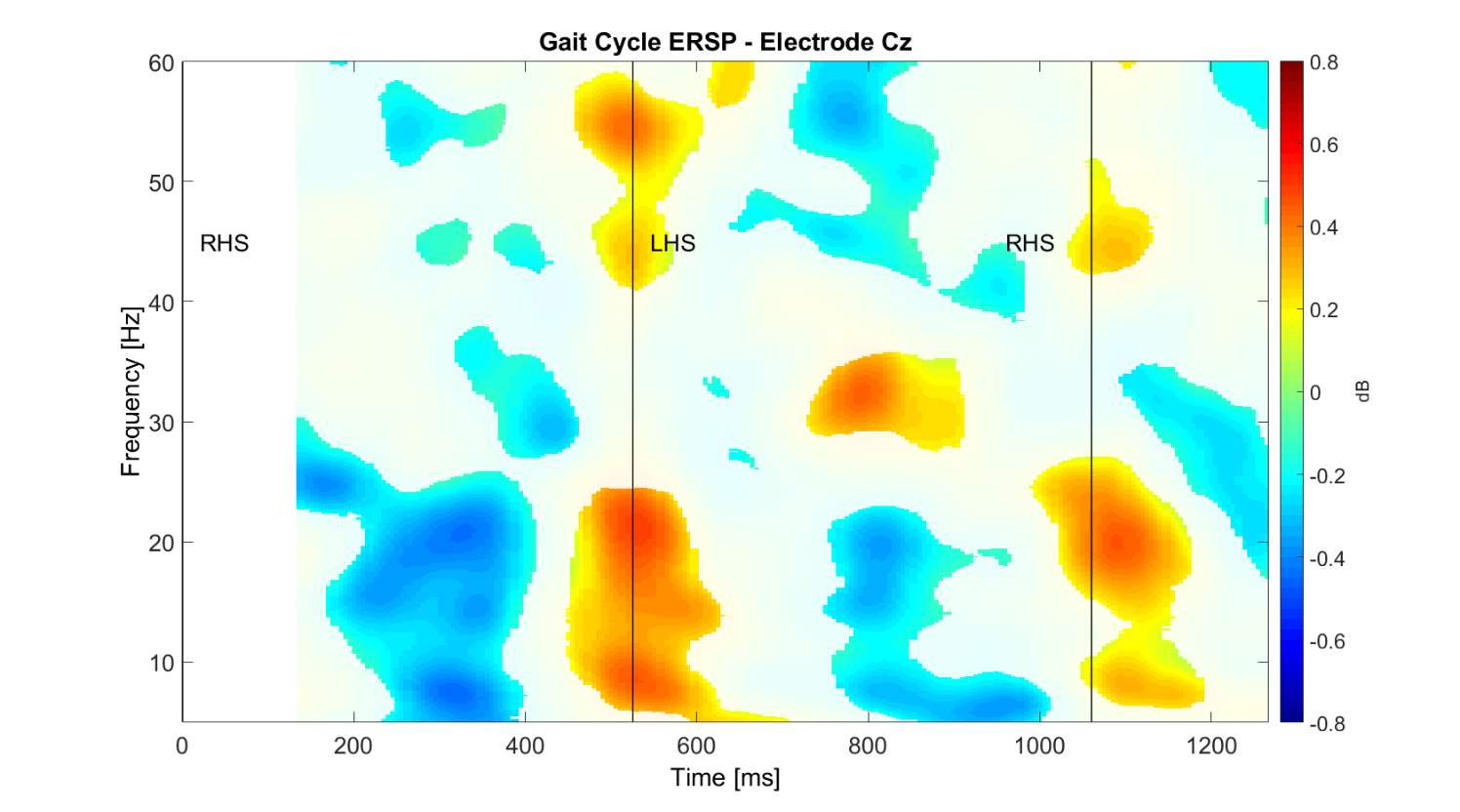
**Supplementary Figure 1.** Single-task walking all subjects (N=14) average ERSPs for Cz electrode, baseline is the log-spectrum of the mean gait cycle. Group-level significance was calculated via a bootstrapping method and FDR correction for multiple comparisons (p < 0.05) according to Wagner et al., 2012. A white mask is applied on those time-frequency bins (i.e. pixels) that did not pass the statistical test. Frequency values ranges from 5 Hz to 60 Hz, which limited the considerable time interval preventing the presentation of a full gait stride.


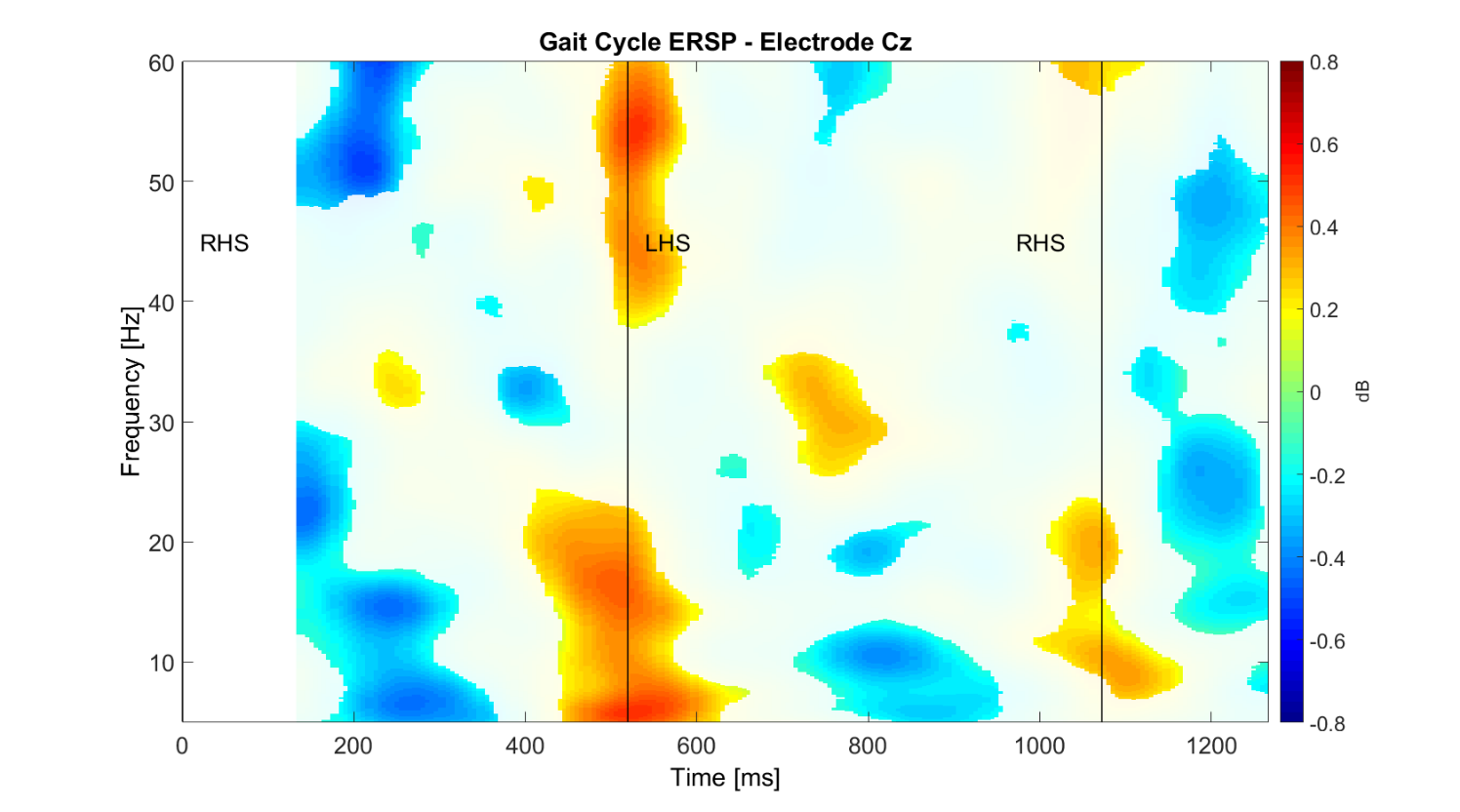


Supplementary Figure 2. Dual-task1 walking (i.e. walking while conversing) all subjects (N=14) average ERSPs for Cz electrode, baseline is the log-spectrum of the mean gait cycle. Group-level significance was calculated via a bootstrapping method and FDR correction for multiple comparisons (p < 0.05) according to Wagner et al., 2012. A white mask is applied on those time-frequency bins (i.e. pixels) that did not pass the statistical test. Frequency values ranges from 5 Hz to 60 Hz, which limited the considerable time interval preventing the presentation of a full gait stride.


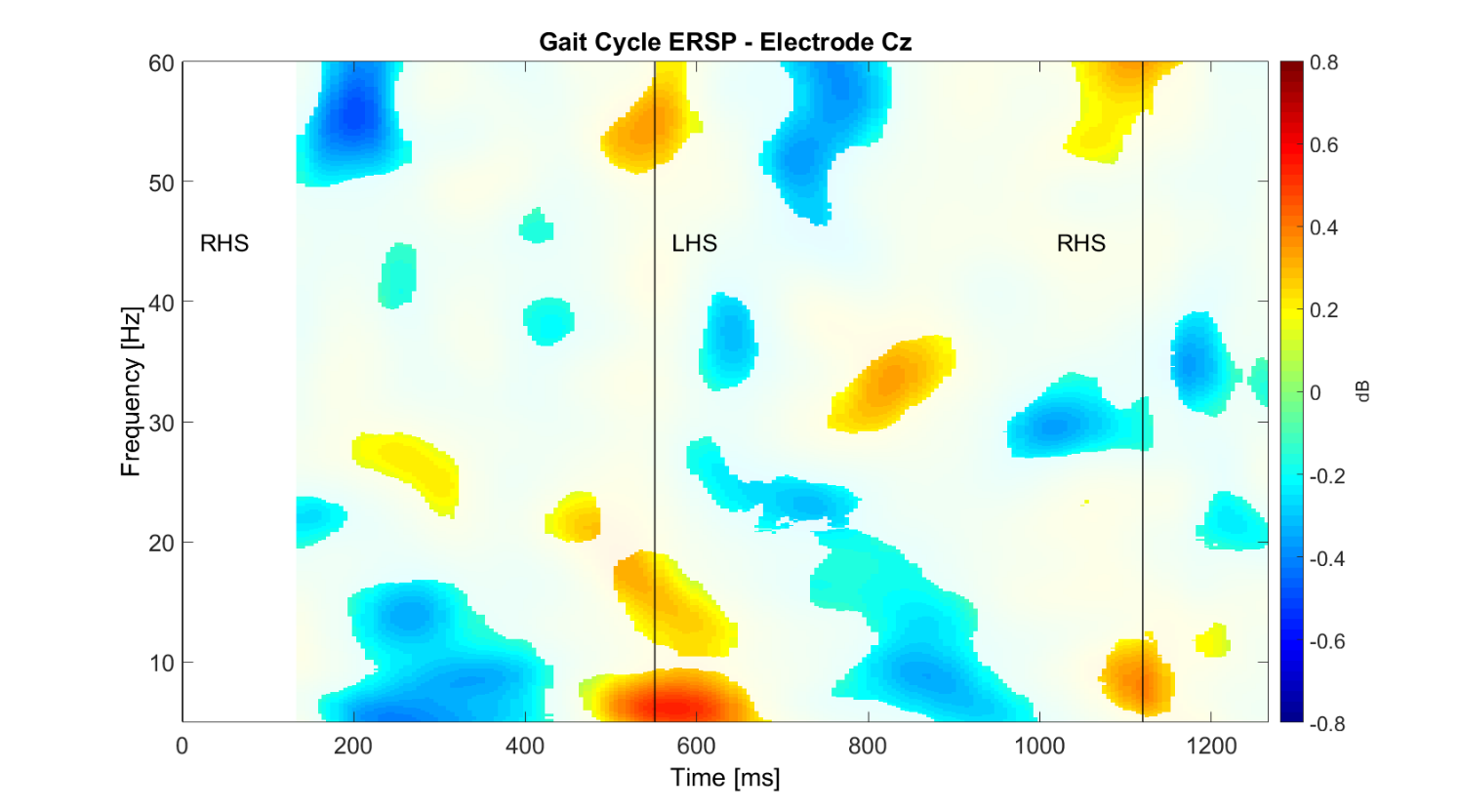


Supplementary Figure 3. Dual-task2 walking (i.e. walking while texting with a smartphone) all subjects (N=14) average ERSPs for Cz electrode, baseline is the log-spectrum of the mean gait cycle. Group-level significance was calculated via a bootstrapping method and FDR correction for multiple comparisons (p < 0.05) according to Wagner et al., 2012. A white mask is applied on those time-frequency bins (i.e. pixels) that did not pass the statistical test. Frequency values ranges from 5 Hz to 60 Hz, which limited the considerable time interval preventing the presentation of a full gait stride.

**S2 - Time-frequency analyses on electrodes close to face and neck**


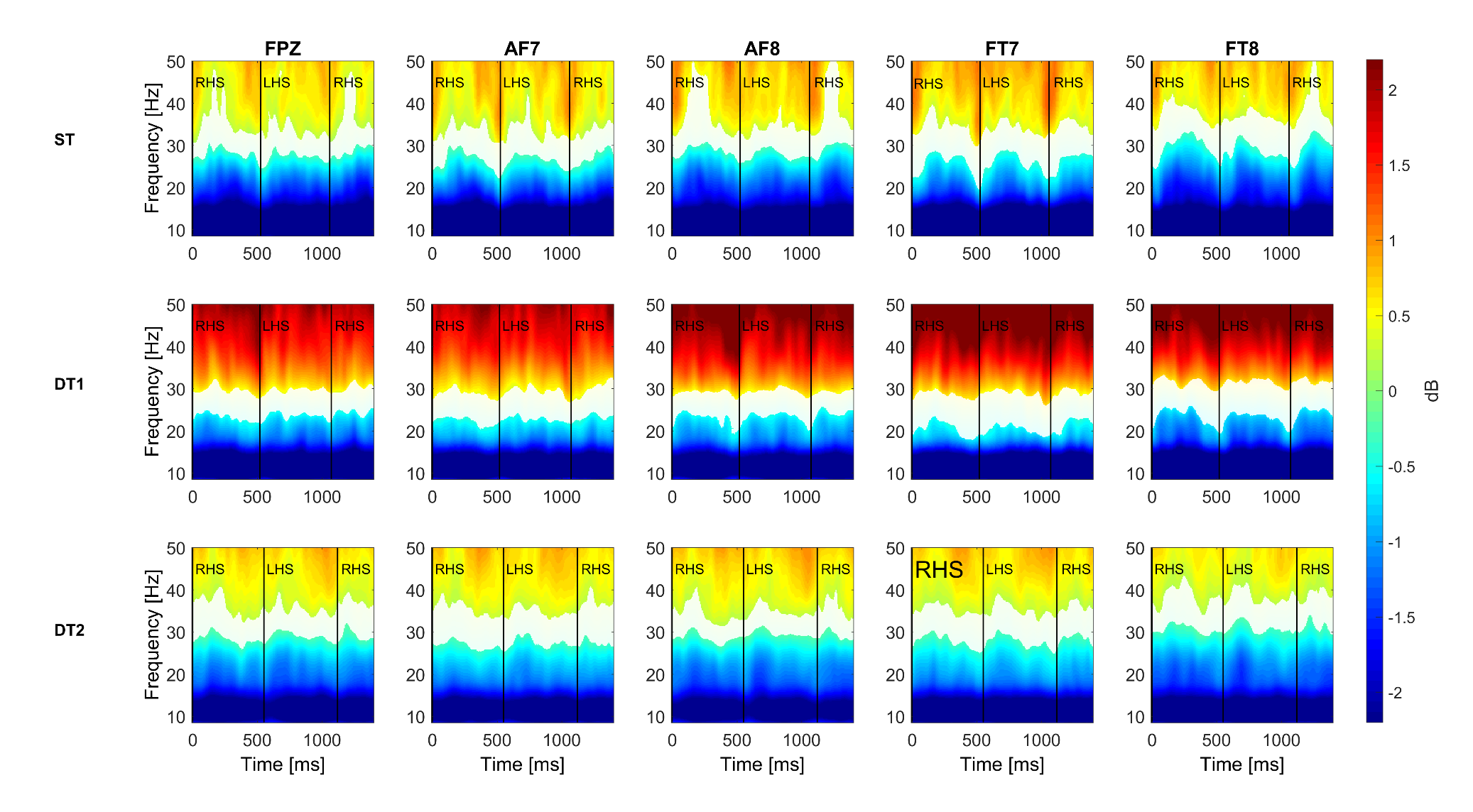


Supplementary Figure 4. Group-level time-frequency analyses across conditions for frontal electrodes with resting state baseline. All subjects (N=14) average time (x axis) – frequency (y axis) representations of the spectral power of frontal electrodes are here reported for each condition (first row: single-task walking; second row: dual-task1 walking; third row: dual-task2 walking). The log spectrum of a 3 minutes period of resting state standing still with eyes-open was used as baseline. Colour-bars (dB) are constant across conditions and report increase (values > 0, warm-colour coded) and decrease (values < 0, cold-colour coded) of power spectrum with respect to the baseline. Group-level significance was calculated via a bootstrapping method and FDR correction for multiple comparisons (p < 0.05) according to Wagner et al., 2012. A white mask was applied on those time-frequency bins (i.e. pixels) that did not pass the statistical test.


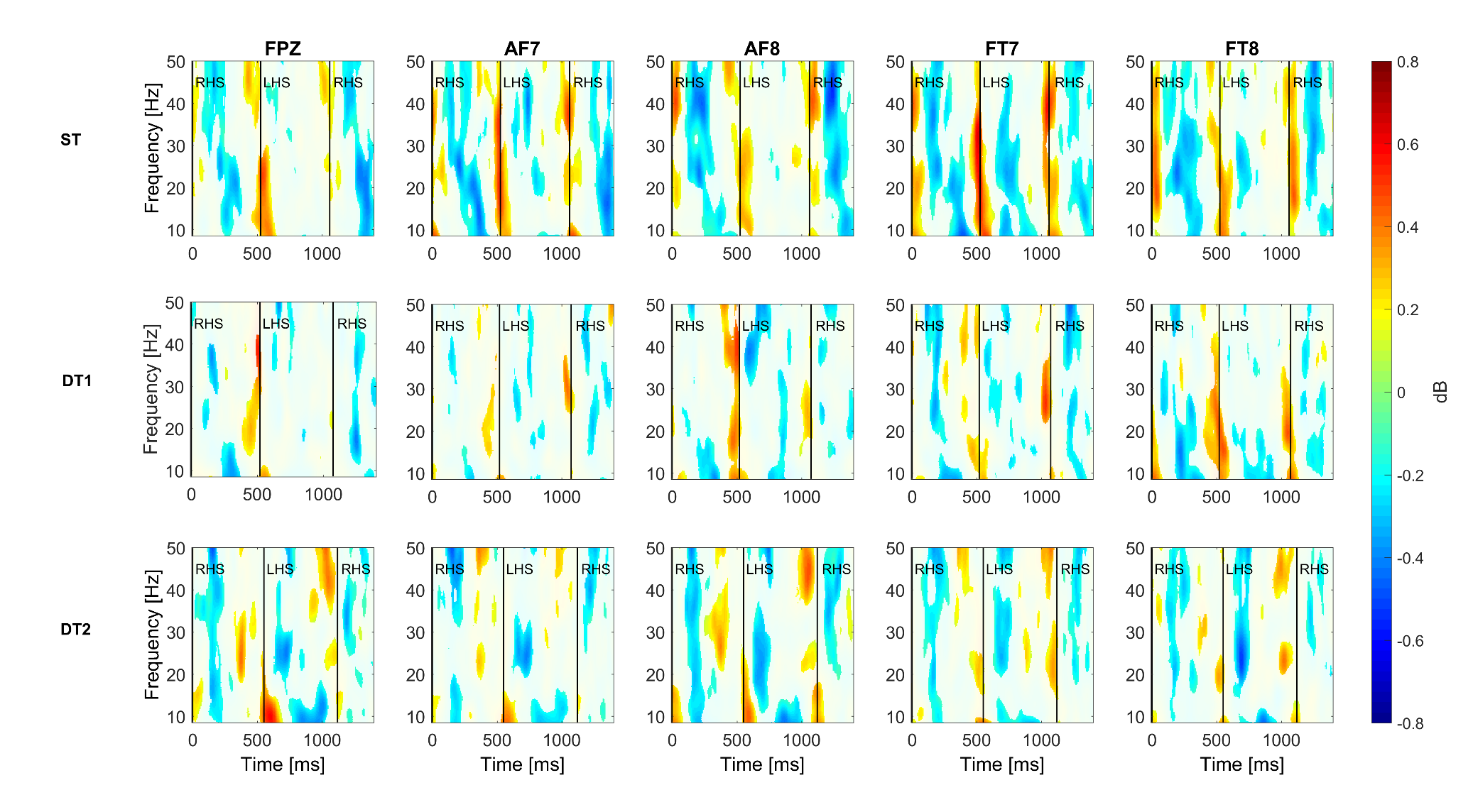


Supplementary Figure 5 - Group-level time-frequency analyses across conditions for frontal electrodes with mean gait cycle baseline. All subjects (N=14) average time (x axis) – frequency (y axis) representations of the spectral power of frontal electrodes are here reported for each condition (first row: single-task walking; second row: dual-task1 walking; third row: dual-task2 walking). The mean gait cycle log spectrum was employed as baseline. Colour-bars (dB) are constant across conditions and report increase (values > 0, warm-colour coded) and decrease (values < 0, cold-colour coded) of power spectrum with respect to the baseline. Group-level significance was calculated via a bootstrapping method and FDR correction for multiple comparisons (p < 0.05) according to Wagner et al., 2012. A white mask was applied on those time-frequency bins (i.e. pixels) that did not pass the statistical test.


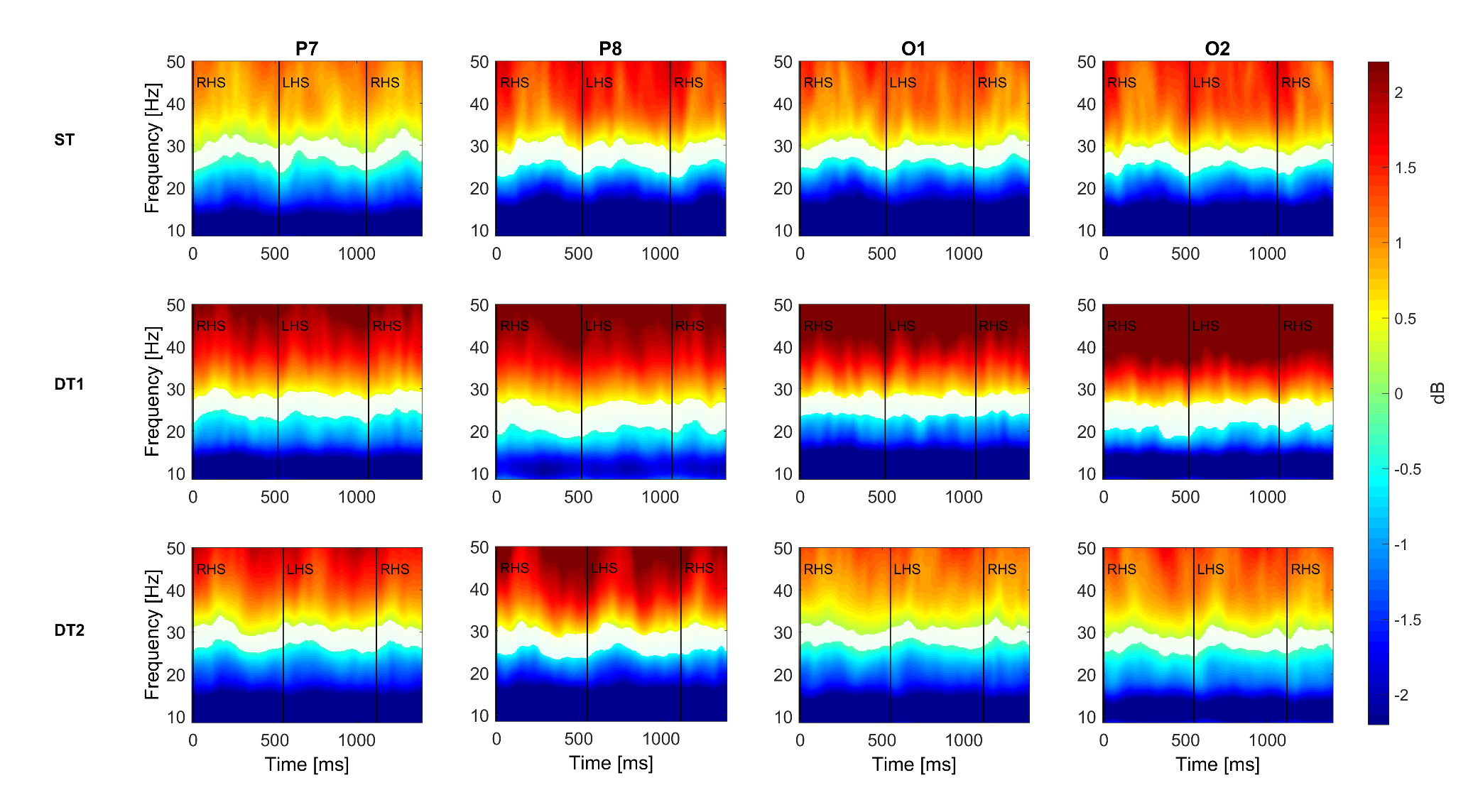


Supplementary Figure 6 - Group-level time-frequency analyses across conditions for posterior electrodes with resting state baseline. All subjects (N=14) average time (x axis) – frequency (y axis) representations of the spectral power of posterior electrodes are here reported for each condition (first row: single-task walking; second row: dual-task1 walking; third row: dual-task2 walking). The log spectrum of a 3 minutes period of resting state standing still with eyes-open was used as baseline. Colour-bars (dB) are constant across conditions and report increase (values > 0, warm-colour coded) and decrease (values < 0, cold-colour coded) of power spectrum with respect to the baseline. Group-level significance was calculated via a bootstrapping method and FDR correction for multiple comparisons (p < 0.05) according to Wagner et al., 2012. A white mask was applied on those time-frequency bins (i.e. pixels) that did not pass the statistical test.


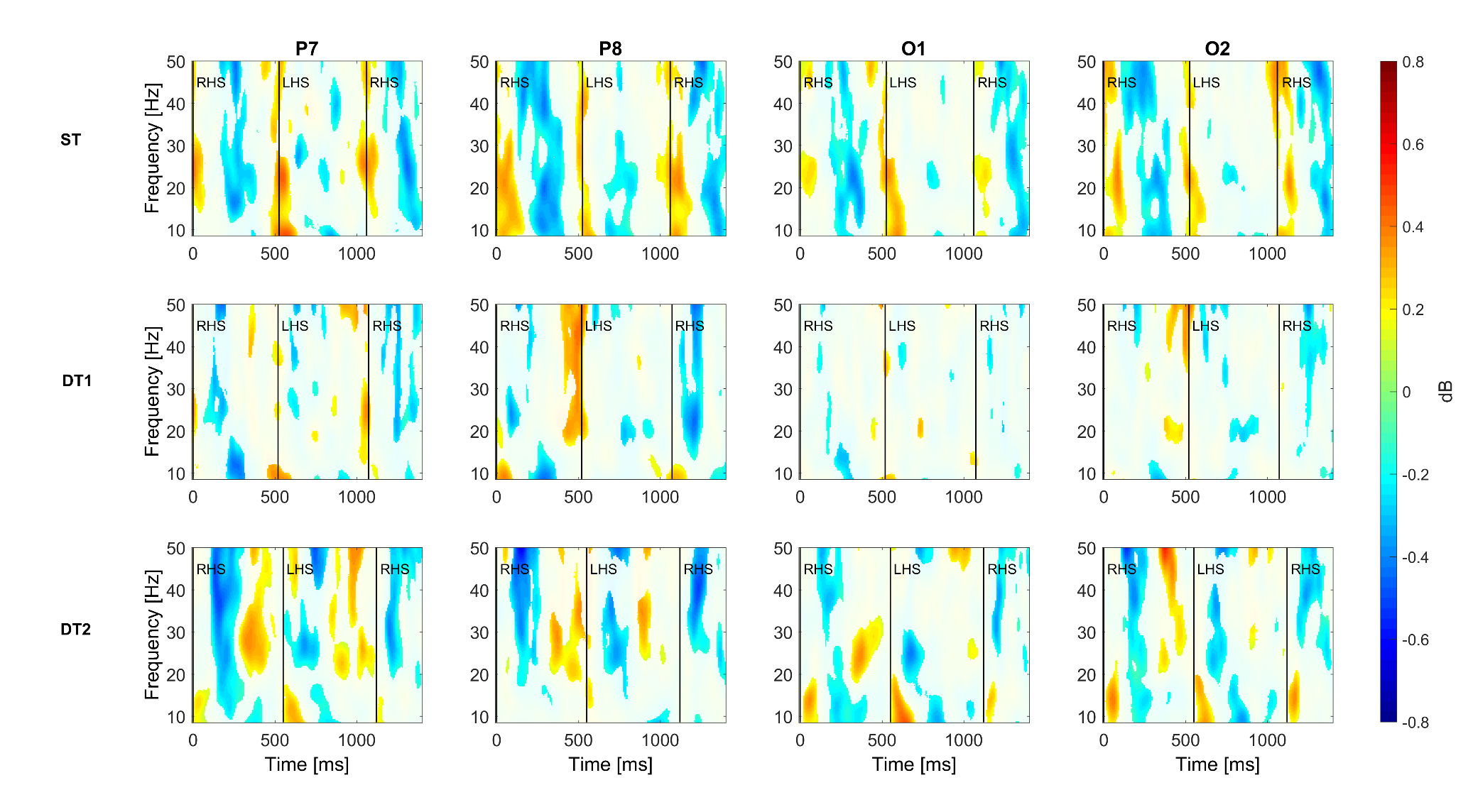


Supplementary Figure 7 - Group-level time-frequency analyses across conditions for posterior electrodes with mean gait cycle baseline. All subjects (N=14) average time (x axis) – frequency (y axis) representations of the spectral power of posterior electrodes are here reported for each condition (first row: single-task walking; second row: dual-task1 walking; third row: dual-task2 walking). The mean gait cycle log spectrum was employed as baseline. Colour-bars (dB) are constant across conditions and report increase (values > 0, warm-colour coded) and decrease (values < 0, cold-colour coded) of power spectrum with respect to the baseline. Group-level significance was calculated via a bootstrapping method and FDR correction for multiple comparisons (p < 0.05) according to Wagner et al., 2012. A white mask was applied on those time-frequency bins (i.e. pixels) that did not pass the statistical test.
